# A Quick “Environment Check” for All Ages: Validating the New Age-Inclusive Work Environments Instrument

**DOI:** 10.1093/geroni/igac066

**Published:** 2022-10-27

**Authors:** Mikaela S Owen, Hanne Berthelsen, Stephanie D Jamieson, Hugo Westerlund

**Affiliations:** Behaviour–Brain–Body Research Group & Psychosocial Safety Climate Global Observatory, Justice & Society, University of South Australia, Adelaide, Australia; Centre for Work Life and Evaluation Studies (CTA) & Faculty of Odontology, Malmo University, Malmo, Sweden; Centre for Workplace Excellence, Business School, University of South Australia, Adelaide, Australia; Stress Research Institute, Stockholm University, Stockholm, Sweden

**Keywords:** Aging workers, Development opportunities, Discrimination, Inclusion, Scale validation

## Abstract

**Background and Objectives:**

The global aging workforce necessitates new approaches in designing work environments to cater to the needs of increasingly age-diverse work groups. The Organisation for Economic Co-operation and Development (OECD) has in reaction outlined that organizations need to provide age-inclusive work environments that support the needs of their multigenerational workforce, to ensure their sustainability and profitability. To capture the age inclusiveness of the work environment, the present study proposes and validates an age-inclusive “environment check” for organizations referred to as the Age-Inclusive Work Environment Instrument (AIWEI), which covers discrimination, inclusion, and development opportunities.

**Research Design and Methods:**

We validate the 9-item AIWEI using cross-sectional and multilevel data from 2,892 Swedish workers across 101 workplaces who completed an online survey, using confirmatory factor analyses across young, middle-age, and older workers. Using a nomological approach, we also evaluate the concurrent validity of the AIWEI with a 2-1-1 path analysis.

**Results:**

The factor analyses supported a 3-factor model comprising of inclusion, discrimination, and development opportunities, across 3 age groups (i.e., young, middle-age, and older workers). These 3 factors had high Intraclass Coefficient (ICC) scores showing consistency in responding in the workplace. In accordance with the nomological approach, the factors of the AIWEI were linked with Psychosocial Safety Climate, burnout, and engagement, demonstrating concurrent validity for the AIWEI.

**Discussion and Implications:**

This new “environment check” provides a way to capture age-inclusive work environments for both younger and older workers, in an age-diverse workforce. In the validation process, age-inclusive work environments were found to exist as a group phenomenon, through shared perceptions within an organization, as well as an individual phenomenon, as experiences specific to an individual. This is important for the development and implementation of policies and strategies designed to benefit workers and organizations.

**Translational Significance:** We proposed an instrument capturing age-inclusive work environments as experienced by both younger and older workers in age diverse work groups; a useful feature in increasingly age-diverse workforces. The instrument can act as a quick check for tracking organizations’ performance, and more broadly, benchmarking to identify, manage, and monitor age-inclusive work environment factors at the national level. The instrument can be used with lengthier risk assessments, such as the Copenhagen Psychosocial Questionnaire III, to glean comprehensive understandings of the psychosocial work environment, for the betterment of workers’ and organizations’ outcomes, in the context of aging work populations.

The workforce is aging across the globe (OECD, 2020); a shift which is actively encouraged by governments to reduce the burden on their social welfare system and to retain skilled and knowledgeable labor (Phillips & Siu, 2012; Sewdas et al., 2017). However, an aging workforce gives rise to new challenges in the workplace. One of the challenges faced by organizations is providing environments that meet the needs of a multigenerational workforce to ensure the sustainability and profitability of their business in this new age-diverse working climate (OECD, 2020). To address this challenge there is a budding area of research on age-inclusive workplaces, framed as age-friendly workplaces, that addresses the organizational factors rather than placing the burden on the workers to adapt to the working conditions (Eppler-Hattab et al., 2020b). An Age-Friendly Workplace refers to the extent in which “older employees are able to know and feel that they are accepted and treated according to their competencies and needs” (Eppler-Hattab et al., 2020b, p. 14). To capture the conceptualization of age inclusiveness, based on Age-Friendly Workplaces, we propose a new instrument; the Age-Inclusive Work Environment Instrument (AIWEI), that considers the psychosocial work environment as experienced by younger and older workers, in an age diverse workplace. That is, we expand the conceptualization of age inclusiveness to incorporate both younger and older workers to address the increasingly age diverse workforce, and to measure this more comprehensive conceptualization of age inclusiveness with the AIWEI. As such, the aim of the current paper is to propose and validate the AIWEI that captures the age inclusiveness within the workplace in terms discrimination, inclusion, and opportunities for development for both younger and older workers in an age-diverse workplace.

## Age-Inclusive Work Environments

Despite the importance of adapting to age-diverse populations in the workplace, little research has been conducted on how to measure age inclusiveness from the perspective of age-friendly work environments. The bulk of the literature exploring age-friendly work environments to understand age inclusiveness have highlighted best practice or discussed age-inclusive factors through discussion papers or case studies (Appannah & Biggs, 2015; Broughan, 2013; Ciampa & Chernesky, 2013). Limited research has measured worker perceptions of age-inclusive work environments. Additionally, the research into age-inclusive work environments has largely focused on older workers to the exclusion of younger workers.

Providing age-inclusive work environments is an important step in addressing the increasingly age-diverse work groups that are seen in workplaces across the globe (OECD, 2020). If appropriately designed, an age-inclusive workplace with an age-diverse work group can enhance workers’ productivity, performance, and innovation (Backes-Gellner & Veen, 2013; Boehm et al., 2014). The original conceptualizations of age-friendly work environments did not take a life-span approach, as they largely focused on the needs of older workers (Appannah & Biggs, 2015; de Guzman et al., 2014; Eppler-Hattab et al., 2020b). However, we are at a unique point in time where organizations are facing the challenge of having up to five different generations working alongside each other in the same workplace (Windham-Bradstock, 2021). As such, it is important to expand this conceptualization of age friendliness to incorporate younger workers so that age inclusiveness considers the needs across the life span, and consequently addresses the challenges and needs of the multigenerational workforce. For example, younger workers are more susceptible to nonfatal injuries and higher levels of organizational movements (turnover), while older workers are more susceptible to fatal injuries, as well as early retirement (Billet et al., 2011; Grosch & Scholl, 2020; Maurer et al., 2008; Silverstein, 2008; Topf, 2000). Therefore, the goal of age-inclusive work environments is to design workplaces and work tasks that ensure the health and productivity of workers (Grosch & Scholl, 2020) across their working life.

As previously discussed, age-inclusive work environ-ments refer to the extent workers feel accepted and are treated based on their competencies and needs rather than their chronological age (Eppler-Hattab et al., 2020b). Consistent with this definition, there are three core psychological factors often highlighted in the age-inclusive literature based on age friendliness, discrimination, a sense of inclusion, and opportunities for development (Appannah & Biggs, 2015; Broughan, 2013; Ciampa & Chernesky, 2013; Eppler-Hattab et al., 2020a; Paullin & Whetzel, 2012). These three psychological factors—discrimination, inclusion, and opportunities for development—align with the three core psychological needs universally outlined by the Self Determination Theory (SDT): relatedness, autonomy, and competence (Deci & Ryan, 2000). In accordance with the SDT, individuals can flourish when all three needs are met in terms of development and well-being; however, when even one need is not met an individual will experience a degradation in psychological growth and ill-being (Deci & Ryan, 2000). In terms of the proposed AIWEI, discrimination acts as a barrier for the needs of *relatedness* and *autonomy. Relatedness* refers to the need to feel connected to others, such as colleagues (Deci & Ryan, 2000), while *autonomy* refers to the need to be true to oneself in terms of behavior and experience (Deci & Ryan, 2000). As such, in the presence of discrimination in which individuals may feel disconnected to others and may not be able to act in a way that is true to themselves, the need for *relatedness* and *autonomy* can undermine the workers’ development and wellbeing. Inclusion also reflects the needs *relatedness* and *autonomy*. Inclusion provides workers with the sense that they belong and simultaneously protecting and enhancing their sense of uniqueness (Shore et al., 2011). That is, their sense of belonging and their sense of being true to themselves. Hence, inclusion provides workers with their need for *relatedness* with the sense of belonging and the need for *autonomy*, with the freedom to be unique and true to themselves. As such, discrimination acts as the barrier to having the needs of *relatedness* and *autonomy* being met while inclusion acts as the enabler. Finally, opportunities for development reflects the need for *competence*, in which workers feel they are having a positive effect on their environment and feel they can meet the outlined challenges and goals among other valued outcomes (Deci & Ryan, 2000; Osemeke & Adegboyega, 2017). Opportunities for development can provide workers with skills and knowledge required to meet the new challenges and hence need for *competence* in a fast-moving and constantly changing world of work. Therefore, we propose the Age-Inclusive Work Environments Instrument (AIWEI) that encompasses these three core elements: discrimination, inclusion, and development, to measure age-inclusive work environments for older and younger workers in an age-diverse workplace.

### Age Discrimination

Age discrimination, also referred to as ageism, can be understood as the “process of systematic stereotyping and discrimination against people because they are old” (Butler, 1975). Direct discrimination is the disadvantage an individual experiences due to unfair treatment as result of their personal characteristics, such as age (Government Offices of Sweden, 2015). Indirect discrimination, on the other hand, is the implementation of policies, practices, or procedures that on the surface appear neutral but have the potential to disadvantage people based on factors such as age, when the “neutral” policies, practices, or procedures are inappropriately enforced.

Age discrimination, whether witnessed in the workplace or experienced directly, has a variety of detrimental consequences for workers’ occupational outcomes. For example, when workers, regardless of age, witness unfair discrimination promotion practices toward older workers in the workplace, they report lower levels of employee engagement (James et al., 2013). Similarly, workers who *experienced* discrimination based on their age reported lower levels of job satisfaction, commitment, and engagement, along with longer sickness absences, and earlier retirement intentions (Bayl-Smith & Griffin, 2014; Macdonald & Levy, 2016; Viitasalo & Nätti, 2015). The impact of age discrimination in the workplace extends beyond the individual to the organization with lowered organizational performance (Kunze et al., 2011).

Much less attention has been given to the experiences of younger workers in the research exploring age discrimination in the workplace. The above-mentioned definition of age discrimination by Butler (1975) was in reference to the discrimination experienced by older individuals. Later conceptualizations of age discrimination acknowledged that younger workers can also be the target of this form of discrimination (Iversen et al., 2009). Despite the inclusion of all ages into the conceptualization of age discrimination only a small amount of research has been done among young individuals, particularly in the workplace (Chasteen et al., 2020; Snape & Redman, 2003). This lack of research is of concern, as young workers can also experience age discrimination in the workplace (Chasteen et al., 2020). Some of the stereotypes that are targeted at younger workers are, for example, that they are lazy and irresponsible (Finkelstein et al., 2013). In contrast, the stereotypes that tend to be directed at older workers are that, while experienced, they are also resistant to change and unable/unwilling to learn (Finkelstein et al., 2013).

### Inclusion in the Workplace

Diversity in organizations, in terms of age, gender, and race, is increasingly becoming the norm due to increasingly diverse global populations. However, increasing diversity without ensuring workers experience feelings of inclusion can foster counterproductive outcomes, such as increased task and relationship conflict (Nishii, 2013). Inclusion addresses the need for the integration of diversity in the workplace using processes and systems implemented by the organization (Roberson, 2006). Specifically, inclusion in the workplace refers to “equal opportunity for members of socially marginalized groups to participate and contribute while concurrently providing opportunities for members of non-marginalized groups, and to support employees in their efforts to be fully engaged at all levels of the organization and to be authentically themselves” (Shore et al., 2018, p. 177).

An inclusive work environment builds a sense of belonginess between its workers while simultaneously fostering their uniqueness, regardless of social group status (Nishii, 2013; Shore et al., 2018). That is, organizations that meet the conditions of both belongingness and uniqueness for its workers are high on inclusion across all social groups. Therefore, a workplace that is high on age inclusion is expected to be inclusive of workers’ age status in conjunction with their membership in other diverse/intersecting social groups. If age inclusion is examined in isolation without consideration for intersectionality, it is possible that an exclusionary work context could be misinterpreted as inclusive. For example, a lack of age inclusion could be evident in cases where there is an intersectionality of age with other minority group statuses that hold lower levels of social power, as per the inclusion framework (Shore et al., 2011). That is, in the same working environment, older workers that are both female and Black may report a lack of age inclusion, whereas older White males may conversely report high age inclusion. Therefore, it is important to look beyond the specific domain of age to allow for a multifaceted and richer understanding of experiences at work (McBride et al., 2015).

### Opportunities for Development

To stay relevant in the ever-changing labor market of the 21st century workers need to constantly update their knowledge and skills. Hence, opportunities for development are vital to all workers regardless of age. In the past, it was believed that older workers were “checked out,” “slow to learn,” and “hard to train,” and thus little attention has been paid to their need for development opportunities (James et al., 2011). These negative stereotypes can be implicated in the lack of exposure to the various forms of development opportunities for older employees (Maurer, 2007). There are various ways workers can experience development opportunities outside of the traditional form of formal training. Examples include challenging and complex work assignments, job transitions, career counseling, and social networks and relationships (Maurer, 2007). Regardless of the source, older workers were less likely to have access to, or receive encouragement to participate in, various forms of development (Maurer, 2007).

Stereotypes about older workers having a lack of interest in development opportunities are currently being challenged (Henry et al., 2015). For example, when older workers report increased development opportunities, their turnover intentions are correspondingly lower (Henry et al., 2015). Reduced turnover intentions and higher commitment to the organization are just a few recognized outcomes connected to development opportunities (Henry et al., 2015; Tansky & Cohen, 2001), which keep older workers’ built-up industry and occupational experience and knowledge in the organization. In addition, increasing workers development opportunities has also been linked with increased career and job satisfaction (Lee & Bruvold, 2003; Maurer & Chapman, 2013).

## Age-Inclusive Work Environments in Context of the Psychosocial Safety Climate-Extended Job Demands Resources Model

The concurrent validity of the age-inclusive work environments instrument was tested by examining the relationship between this instrument and other measures of related concepts. Specifically, the Psychosocial Safety Climate (PSC; Dollard & Bakker, 2010) extended Job Demands Resources Model (JD-R; Dollard & Bakker, 2010) was used as a nomological framework to guide the validation assessment for the AIWEI.

In the PSC-extended JD-R model, PSC acts as an upstream antecedent of psychosocial risk factors in the workplace, and workers’ health and organizational outcomes, through the health erosion and motivational pathways, respectively (Dollard & Bakker, 2010; Owen et al., 2016; Zadow et al., 2019). PSC refers to the developed and enacted policies, practices, and procedures for the protection of the workers’ psychological health and safety in an organization (Dollard & Bakker, 2010), and is often conceptualized at the group level reflecting the shared perceptions of workers in the same workgroup or organization. In organizations with higher PSC, workers report lower job demands and better access to resources (Owen et al., 2016; Zadow et al., 2019), which in turn promotes better health outcomes (e.g., lower symptoms of depression, psychological distress, and emotional exhaustion), and organizational outputs (e.g., higher worker engagement and performance, fewer injuries, and greater job satisfaction; Owen et al., 2016; Zadow et al., 2019). As there is established support for the impact of PSC on psychosocial risks in the work environment, it is anticipated that PSC will act as a leading indicator for the three psychosocial factors of the AIWEI and the consequent health and motivational outcomes of engagement and burnout.

Examining the relationship between group perceived PSC and individually perceived age-inclusive work environments provide an opportunity to test the concurrent validity of the proposed instrument. That is, the shared perspectives of PSC between workers in the same organization onto their individual experiences of age inclusivity in their workplace. Specifically, we test a proposed model of PSC in the workplace to the employees’ perception of inclusion, development opportunities, as well as discrimination, and consequently burnout and engagement.

## Previous Instruments for Measuring Age-Inclusive Work Environments

Few instruments are available that measure age-inclusiveness in the workplace in relation to age-friendliness. Contributing to the limited research measuring age inclusive work environments from the age-friendliness perspective are the Workplace Age-Friendliness Measure (WAFM) by Eppler-Hattab et al. (2020a) and the unnamed instrument developed by de Guzman et al. (2014). The WAFM is a 24-item scale covering four indicators of an age-friendly organizational climate: age-friendly core culture, development, wellness, and flexibility. Although the WAFM is comprehensive, shorter, more easily administered scales may be beneficial for providing quick “environment checks” of age-inclusive work environments, for use in workplace surveys and epidemiological studies. The WAFM also focuses exclusively on older employees and is only suitable for workplaces where older workers have sufficient representation in their work groups (Eppler-Hattab et al., 2020a). Consequently, a perspective that is inclusive of every age group has been proposed as a possible avenue of enquiry by the developers of the WAFM (Eppler-Hattab et al., 2020a).

The short 11-item instrument provided by de Guzman et al. (2014) captures both the physical and psychological aspects of age-inclusive workplace environments. However, consistent with the WAFM, this instrument by de Guzman et al. (2014) does not directly capture age-inclusive work environments for both younger and older workers. Instead, the instrument has been designed for older workers’ and their experience of the age-inclusive work environment. Additionally, while the instrument is easy to use and understand, the items are measured with dichotomous response options that have little variability in responses due to the items picking up on very high-frequency events, such as nonspecific social programs and trainings for technologies (de Guzman et al., 2014). As such, this short and easy-to-use instrument does not capture the extent of availability or exposure to the important aspects needed for an age-inclusive workplace environment. To address this need for an instrument that can be used for younger and older workers, we propose a short-form instrument that captures the level of exposure to age-inclusive factors in the work environment with mixed age groups.

## Current Study

The aim of the current study is to develop and validate an instrument that captures age-inclusiveness in the workplace for age-diverse work groups in terms of inclusion, opportunities for development, and lack of discrimination, for both younger and older workers. More specifically, we (a) evaluate the construct validity of the AIWEI and its measurement invariance across different age groups; (b) test the appropriateness of aggregating individual-level responses to the workplace level; and (c) evaluate the concurrent validity of the AIWEI using the nomological network of the PSC-extended JD-R model.

## Method

### Participants

Cross-sectional questionnaire data were collected in 2017–2020. In collaboration with occupational health services and other occupational and safety stakeholders, workplaces could sign up for a work environment survey followed by a report with their own results as part of the development and research project. This led to a convenience sample of 101 workplaces. All staff members at these workplaces received an e-mail with a link to an online questionnaire and information about the research project. We defined workplace as geographically separate units where people conduct their daily work and share the same local management. Each survey was open 3–4 weeks and included two reminders. The average response rate for the sample of individuals within the workplaces was 79% (2,892 non-managerial respondents). The anonymity of participants and the workplaces included were maintained throughout the study.

In the current study, data on 2,892 Swedish workers in which the majority were female (63.7%), age between 35 and 54 (49.8%) was included. During a typical work week, most workers in the current sample worked between 31 and 40 hr, with nearly 9 in 10 workers having direct contact with an end-user (e.g., patient, customer, or client). The industries the workers predominately worked in included public administration and defense/compulsory social insurance, health and social care/social service, training, and transport and storage.

The demographic composition was mostly consistent across the three age groups: younger workers (18–34), middle-aged workers (35–54), and older workers (55 and above; Supplementary Table 1). Differences can be seen between the age groups for hours worked and industry of employment. The pattern indicates that a higher proportion of workers in the younger age group were working over 40 hr than the middle-age and older workers. In terms of industry, there was a higher proportion of younger workers in the public administration and defense/compulsory social insurance than middle-age and older workers. In contrast, there were a higher proportion of middle-age and older workers in the training industry than younger workers. Similarly, there were a higher proportion of middle-age and older workers in the health and social care/social services than younger workers.

To account for the multigroup analyses by age group, an inclusion criterion of workplaces where at least 10 respondents had filled in the questionnaire was applied for multilevel analyses with individuals aggregated to the workplace (2,744 individuals nested in 82 workplaces). That is, to ensure sufficient age diversity in each workplace and the reliability of group mean scores (Bliese, 1998), only workplaces where at least 10 respondents had filled in the questionnaire were included in the multilevel analyses. This led to a subsample of 2,744 individuals nested in 82 workplaces.

### Measures

#### Age-Inclusive Work Environment

The AIWEI is a nine-item instrument that measures workers’ perceptions of inclusion, development opportunities, and discrimination. From the nine items that compromise the AIWEI, three items were selected from the Nordic Questionnaire for Monitoring the Age-Diverse Workplace (QPSNordic-ADW; Pahkin et al., 2008) and one item developed through expert discussion. All four items were tested in cognitive interviews in connection to the Swedish validation study of the Copenhagen Psychosocial Questionnaire II (COPSOQ II). One item for the AIWEI was directly from the QPSNordic-ADW (Pahkin et al., 2008) and addresses inclusion in the workplace “Are older workers’ experience appreciated at your workplace?” An additional item on age inclusiveness was developed through topic expert discussions on the push and pull factors in the workplace in terms of generativity. A further two items on age discrimination were adapted from the QPSNordic-ADW, in which two items were derived from a single item; “Have you noticed any inequalities in how older and younger workers are treated at your workplace?” Example items include “These questions are about whether there is room for everyone in your workplace—Are the experiences of older people appreciated in your workplace?” and “Have you noticed that younger employees are discriminated against in your workplace?”

The five remaining items of the AIWEI are from the COPSOQ, which is a generic instrument bridging workplace risk assessments and research on work and health (Burr et al., 2019). Of the five remaining items, three measure possibilities for development from the Swedish standard version of COPSOQ III (Berthelsen et al., 2020), and two capture social inclusiveness from the Swedish version of COPSOQ II (Berthelsen et al., 2014; Pejtersen et al., 2010) An example item is “These questions are about yourself in relation to your work tasks—Does your job offer opportunities to develop your skills?” All nine items had five response alternatives: To a very large extent, To a large extent, Somewhat, To a small extent, To a very small extent, with scores ranging from 0 to 100 in accordance with the COPSOQ tradition (Berthelsen et al. 2014; Burr et al., 2019). See Supplementary Table 2 for a full list of items in English and Swedish.

#### Psychosocial Safety Climate

PSC was measured using the shortened four-item Psychosocial Safety Climate scale (Berthelsen et al., 2020; Hall et al., 2010). Items were measured using a 5-point Likert scale, ranging from 1 “Strongly disagree” to 5 “Strongly agree.” Higher scores indicate higher levels of PSC and hence represent a lower risk to workers’ psychological health and safety. An example item includes “Senior management shows support for stress prevention through involvement and commitment.”

#### Burnout and engagement

The burnout and engagement subscales used in the current study were drawn from the COPSOQ III (Berthelsen et al., 2020; Burr et al., 2019; Schaufeli et al., 2002). To measure workers’ level of burnout, participants responded to four items, and three items for engagement. The items were measured on a 5-point Likert scale with scores ranging from 0 “A very small extent” to 100 “To a very large extent.” An example item of burnout is “How often have you been emotionally exhausted?” An example item of engagement is “At my work, I feel bursting with energy.”

#### Aggregation

Included in analysis were 2,744 Swedish workers clustered into 82 workplaces, after excluding those who had less than 10 workers from their organization respond to the survey. The average size of the workplace was 33.46 workers (Min = 10, Max = 138). ICC1 scores represent the amount of variance that can be explained by group memberships (e.g., employees within a workplace; Bliese et al., 2019; LeBreton & Senter, 2007). ICC1 scores above 0.05 reflect a small to medium effect, and higher values indicating stronger effects (LeBreton & Senter, 2007). ICC2 is an estimate of the reliability based on group membership (Bliese et al., 2019; LeBreton & Senter, 2007). ICC2 scores <0.50 reflect poor reliability, 0.5–0.75 moderate, and >0.75 good reliability for responses based on group membership (Koo & Li, 2019).

### Analysis

#### Confirmatory factor analysis

In AMOS v26 a confirmatory factor analysis (CFA) with maximum likelihood estimation was run to test the model fit for the proposed age-inclusive work environments instrument. Model fit was assessed using the χ^2^, Comparative Fit Index (CFI), Standardized Root Mean Square Residual (SRMR), and Root Mean Square Error of Approximation (RMSEA) with the corresponding closeness of fit probability value (PCLOSE). Scores above .95 for CFI indicate adequate fit between the proposed model and the sample data, while scores below 0.05 for SRMR represent adequate fit (Byrne, 2016). Finally, the RMSEA should be below 0.10 with a PCLOSE value > .50 to indicate acceptable model fit (Byrne, 2016).

Building upon the model identified in the CFA, a multi-group confirmatory factor analysis was conducted to test configural, metric, and scalar measurement invariance across the three age groups: younger workers, middle-aged workers, and older workers (Byrne, 2016). Configural invariance assesses if the factor structure is the same across the groups in the multigroup confirmatory factor analysis. Metric invariance assesses the similarity in factor loadings, and finally scalar invariance assesses the equivalence in values/means across the groups. To compare model fit, we used the change in CFI scores, with a cut-off of 0.01, as the χ^2^ is sensitive to large sample sizes (Putnick & Bornstein, 2016).

#### Multilevel path analysis

**Figure 1.**
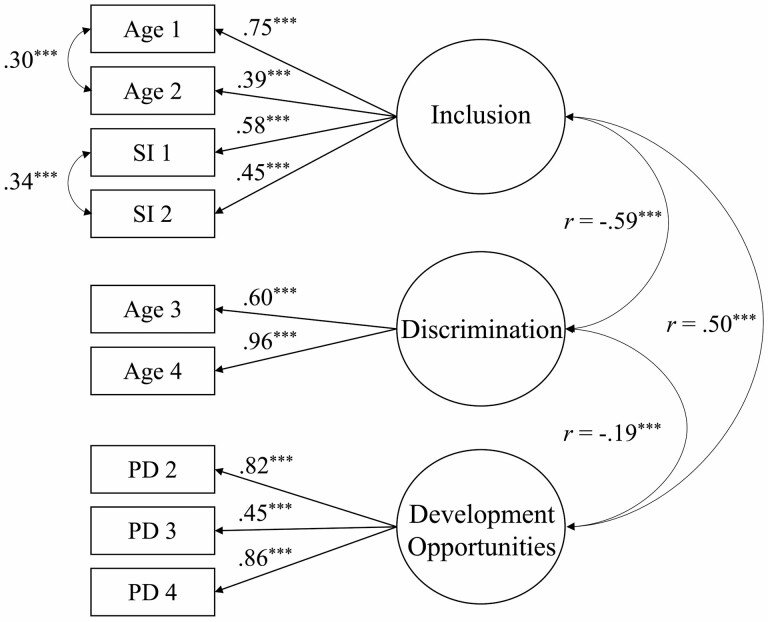
Proposed measurement model of the Age-Inclusive Work Environment Instrument with modifications.

For the test of concurrent validity, a 2-1-1 multilevel path analysis was run in MPLUS v8 using Maximum Likelihood with Robust Errors (MLR) estimation to test the proposed model in Figure 1, while controlling for hours worked and industry. The structure of the data was multilevel with workers (Level 1) nested within workplaces (Level 2). That is, workers’ perceptions of PSC were aggregated to the workplace and modeled at the workplace level (Level 2). Individual perceptions of inclusion, discrimination, development opportunities, engagement, and burnout were modeled at the worker level (Level 1). It was proposed that PSC would directly impact workers’ sense of inclusion, discrimination, and development opportunities, and indirectly impact engagement and burnout.

## Results

The internal consistency for the three dimensions on the AIWEI were acceptable. The Cronbach’s α was 0.71 for inclusion and 0.77 for development opportunities. As discrimination only consisted of two items, a correlation coefficient is used a measure of internal consistency, with a Spearman rank coefficient of 0.64 (*p* < .001), indicating a strong positive correlation.

### Confirmatory Factor Analysis

We tested the CFA measurement model, which specifies the pattern by which each measure loads on a particular factor (Byrne, 2016). The initial model was respecified to allow error covariance between two items that directly measure older workers’ inclusion, and two items that directly measure the sense of equality based on demographics, based on the inspection of the modification indices and their conceptual interrelatedness. That is, the items measuring older workers’ inclusion cover a sense of value through appreciation and mentorship, while the two items both cover equality through gender and ethnicity. The CFA model presented an acceptable fit to the data (χ^2^(22) = 258.06; CFI = 0.96; SRMR = 0.45; RMSEA = 0.064). The final proposed measurement model with factor loadings is shown in Figure 1, with model fit indices found in Table 1.

**Table 1.** Model Fit Indices for the Measurement Model of Age-Inclusive Work Environments as Perceived by Individual Workers

Model	CFI	SRMR	RMSEA	PCLOSE	df	χ^2^	Comparative Model	∆(df) χ^2^	∆CFI
M0. Null Model	0.00	—	0.266	<.001	36	6,685.16***			
M1. Proposed Model	0.91	0.058	0.099	<.001	24	637.05***	M0 v M1	(12)7068.75***	0.91
M2. Proposed Model with Modifications	0.96	0.045	0.064	<.001	22	258.06***	M1 v M2	(2)378.87***	0.05

*Notes*: CFI = Comparative Fit Index; PCLOSE = closeness of fit probability value; RMSEA = Root Mean Square Error of Approximation; SRMR = Standardized Root Mean Square Residual. Modifications included correlating the error terms between Inclusion 2 and Inclusion 3, and the error terms between Inclusion 1 and Inclusion 4.

***p < .001.

### Multigroup Confirmatory Factor Analysis

Using one-way ANOVAs, differences between the three age groups were found for average levels of inclusion (*p* < .001) and discrimination (*p* = .002), but not for development opportunities (*p* = .229). Workers aged under 35 (younger) report significantly higher levels of inclusion than those aged between 35 and 54 (middle-age; *p* < .001) and 55 and over (older; *p* < .001), and workers aged 35–54 years had higher inclusion levels than 55+ (*p* = .038). In terms of discrimination, younger workers had significantly higher levels of discrimination than middle-aged workers (*p* = .004), and older workers (*p* = .020). No differences in discrimination were found between middle-aged and older workers (*p* = 1.00, refer to Table 2).

**Table 2.** Descriptive Statistics in Different Age Groups and Bivariate Correlations Between the Three Factors Exploring Age-Inclusive Work Environment, Along with Burnout and Workers’ Engagement

Variables	ALL		Younger workers		Middle-aged workers		Older workers		1. Inclusion	2. Discrimination	3. Development opportunities	4. Burnout
	*M*	*SD*	*M*	*SD*	*M*	*SD*	*M*	*SD*				
1. Inclusion	69.60	18.08	72.82	16.67	69.00	18.59	66.81	18.09				
2. Discrimination	10.45	16.49	12.19	17.91	9.76	16.13	9.82	15.23	−0.42***			
3. Development opportunities	70.67	18.03	70.98	17.23	70.98	17.95	69.85	19.17	0.35***	−0.22***		
4. Burnout	35.59	25.33	41.26	25.28	35.70	25.09	28.09	24.01	−0.28***	0.26***	−0.23***	
5. Engagement	69.14	20.32	66.85	19.95	70.47	19.71	68.75	21.76	0.34***	−0.20***	0.55***	−0.44***

*Note*s: Scores ranged from 0 to 100 for all factors and outcome variables.

***p < .001.

A multigroup confirmatory factor analysis was conducted to test structural invariance of the instrument. As given in Table 3, young workers (*n* = 725), middle-aged (*n* = 1,286), and older workers (*n* = 573) all reached acceptable model fit, indicating a close fit between the data and proposed model, indicating it is appropriate to continue with our multigroup confirmatory factor analysis checking for measurement invariance.

**Table 3.** Goodness-of-Fit Statistics for Tests of Multigroup Invariance for the Instrument on Age-Inclusive Work Environments, for Young, Middle-age, and Older Workers

Model	CFI	SRMR	RMSEA	PCLOSE	df	χ^2^	Comparative Model	∆(df) χ^2^	∆CFI
Young workers	0.96	0.047	0.063	.064	22	84.64***			
Middle-age workers	0.97	0.043	0.059	.077	22	119.35***			
Older workers	0.95	0.063	0.080	<.01	22	101.77***			
M1. Configural Model	0.964	0.048	0.038	1.00	66	305.76***			
M2. Metric Model	0.960	0.055	0.035	1.00	84	347.30***	M2 v M1	(18) 41.54**	0.004
M3. Scalar Model	0.958	0.054	0.034	1.00	90	365.66***	M3 v M1	(24) 59.90***	0.006

*Notes*: CFI = Comparative Fit Index; PCLOSE = closeness of fit probability value; RMSEA = Root Mean Square Error of Approximation; SRMR = Standardized Root Mean Square Residual. Modifications included correlating the error terms between SI1 and SI2, and the error terms between Age 1 and Age 2.

***p < .001,

**p < .01.

The first and least stringent model, configural model, had acceptable model fit as can be seen in Table 3. Similarly, both the metric invariance and scalar invariance model demonstrate acceptable model fit with the data. As the first step, the configural model was compared to the metric invariance model. The difference in CFI scores did not exceed the 0.01 cut-off to indicate a significant change (*p* < .001). As such, the metric invariance model was not a worse fit to the data. The final model, scalar invariance model was compared with the metric variance in which the CFI change was less than 0.01 demonstrating the scalar invariance was not a worse fit with the data. As such, there is structural invariance across the three groups as anticipated.

### Multilevel Path Analysis

All individual-level factors were significantly bivariate correlated in the expected directions (Table 2). Specifically, higher levels of inclusion and development opportunities, which were moderately positively correlated, corresponded with lower levels of age discrimination and burnout, and higher levels of engagement. Conversely, higher levels of age discrimination corresponded with higher levels of burnout and lower levels of engagement. Finally, engagement and burnout had a moderate negative correlation, in that higher engagement scores coincided with lower levels of burnout. The strongest correlation existed between development opportunities and engagement, with a strong positive correlation. The remaining correlations ranged from weak/small (−0.20) to moderate/medium (−0.44).

All ICC1 scores were significant for the three factors of Age-Inclusive Work Environments—Inclusivity (0.089, *p* < .001), Discrimination (0.132, *p* < .001), and Development Opportunities (0.211, *p* < .001)—along with PSC (0.158, *p* < .001). From the ICC1 scores, we can see that 15.8% of the variance in scores for PSC can be attributed to the group level, and surprisingly 21.1% of the variance in development opportunities can be attributed to organization membership, along with 13.2% of the variance in discrimination. In relation to ICC2, Inclusion (0.763), Discrimination (0.834), and Development Opportunities (0.898) all scored above the 0.75 cut-off for good reliability as anticipated. Similarly, PSC (0.861) also had an ICC2 score above the cut-off of 0.75.

**Figure 2.**
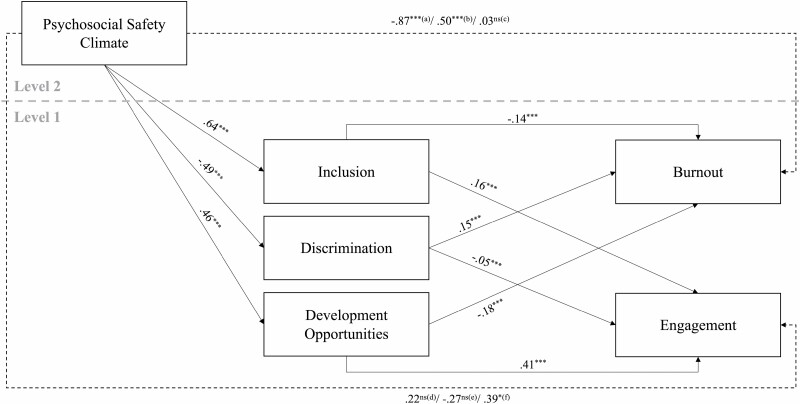
The pathways from Psychosocial Safety Climate (PSC) to Burnout and Engagement through the three factors of the Age-Inclusive Work Environment Instrument controlling for the impact of hours worked and industry upon workers’ level of burnout and engagement. Solid lines indicate direct pathways and broken lines indicated mediated pathways. ^a^PSC to Burnout via Inclusion, ^b^PSC to Burnout via Discrimination, ^c^PSC to Burnout via Development Opportunities, ^d^PSC to Engagement via Inclusion, ^e^PSC to Engagement via Discrimination, ^f^PSC to Engagement via Development Opportunities. Hours controlled for were 31–40 hours. Industries controlled for included Training, and Health and social care/social services. ***p < .001, **p < .01, *p < .05, ^ns^*p* < .05.

The proposed model of AIWEI in its nomological network (Figure 1) was tested for evaluation of concurrent validity. Model fit indices for all workers were 105,235.79 (-2 Log Likelihood (-2LL)), 105,341.53 (Akarke’s Information Criterion (AIC), and 105,559.95 (Schwarz’s Bayesian Criterion (BIC)). As anticipated, group perceived PSC (Level 2) had a positive impact on inclusion and development opportunities, and a negative impact on age discrimination, in the workplace as rated by the individual workers (Level 1), as can be seen in Figure 2. All three dimensions of age-inclusive work environments in turn affected workers’ level of engagement in their workplace, and symptoms of burnout. High levels of inclusion and development opportunities corresponded with high levels of engagement and low levels of burnout. Additionally, increased discrimination in the work environment corresponded to lower levels of engagement and higher levels of burnout.

## Discussion

The purpose of this study was to develop and validate a quick “environment check” instrument for age-inclusive work environments that can be utilized across workplaces irrespective of the age composition of their workforce. We found evidence that our AIWEI is valid for all ages. Specifically, through our process of validation we found support for the three proposed underlying factors—inclusion, discrimination, and development opportunities—which, as anticipated, were associated with PSC, burnout, and engagement within the framework of the PSC-extended JD-R model. Importantly, our instrument demonstrated measurement invariance across the three age groups of workers, in that the dimensions of the AIWEI hold the same meaning for all aged workers. Specifically, the average scores and pattern of responding remained consistent across workers regardless of their age. This consistency is important for capturing age inclusiveness in workplaces that have varying degrees of age diversity among their workers.

In accordance with the hypothesized relationships, based on the logic of the PSC-extended JD-R model, we found that higher levels of PSC corresponded to higher levels of inclusion and development opportunities and lower levels of discrimination. Additionally, higher inclusivity, development opportunities, and lower discrimination were in turn linked with higher engagement and lower burnout. These consistent findings with the literature that PSC is a leading indicator of psychosocial factors in the workplace environment (Dollard & Bakker, 2010; Owen et al., 2016; Zadow et al., 2019) corroborate the concurrent validity of the AIWEI instrument.

### Theoretical Implications

Of interest, we found support for aggregating individual workers’ responses to the workplace level on the three dimensions of the AIWEI: inclusion, discrimination, and development opportunities. These findings indicate that our three factors are measuring something in the workplace at the group level. That is, age-inclusive work environments may exist as a group phenomenon instead of solely reflecting individual experiences of age-inclusive work environments. As such, effecting change in the organization for young and older workers may require efforts not only targeting the individual experiences but also acknowledging and addressing the collective nature of age-inclusive work environments. That is, in addition to one-on-one intervention strategies that target specific individuals or specific groups of individuals, strategies that also develop and strengthen the organizational systems that build inclusion and development opportunities and minimize discrimination should be implemented.

### Practical Implications

A main advantage is that AIWEI can be used as a quick check to track how organizations are meeting their goals for providing an age-inclusive work environment for all their workers, both younger and older, on three key dimensions: inclusion, discrimination, and development opportunities. Previous instrument development has focused on assessing age inclusiveness in relation to older workers and used a lengthier more comprehensive measurement approach (Eppler-Hattab et al., 2020a). In contrast, the AIWEI can be used with workers of all ages and therefore across workplaces with diverse age demographics. This broader applicability may be particularly important for benchmarking efforts to identify, manage, and monitor age-inclusive work environment factors on a national scale. The nine-item length of this instrument provides a shorter option to measure an age-inclusive work environment. This information could be used in practice to further inform a more comprehensive assessment, such as with the 24-item WAFM, to identify the specific areas of concern, among other instruments. This shorter format also reduces the time burden for workers associated with completing the scale.

AIWEI can additionally be used in conjunction with the COPSOQ III to add to the comprehensive psychosocial risk assessment of workplaces for both research and for practice for the betterment of workers’ physical and psychological health, and their organizational outcomes, in relation to the age inclusiveness of the work environment. Notably, the third version of the COPSOQ instrument (Berthelsen et al., 2020; Burr et al, 2019) does not measure discrimination. This omission is important as there are legislative protections for workers in relation to discrimination in Sweden (i.e., the Swedish Discrimination Act; Government Offices of Sweden, 2015) as well as other parts of the world, such as Australia (i.e., Age Discrimination Act 2008). This overlap of items between AIWEI and COPSOQ III means that very little additional time and resources are needed to include age inclusiveness in COPSOQ-based work environment surveys.

### Limitations and Future Directions

The healthy worker effect is a form of selection bias where the actively employed are “healthier” than the general population (Chowdhury et al., 2017). Consistent with this healthy worker effect, it is possible that older workers from non-age-inclusive environments (e.g., low inclusion, high discrimination, and low development opportunities) have tended to retire early and hence were not present in our sample, implicating that most remaining older workers may be in workplaces that are comparatively age-inclusive. Further validation using longitudinal research is warranted, by tracking the participation of older individuals in the workplace to assess if age-inclusive work environments act as a driver for their continued employment.

Using a convenience sample of workplaces is a limitation of the current study. For example, this sampling strategy does not allow for calculating a response rate at workplace level. Additionally, is it unclear if participating workplaces differ from other nonparticipating workplaces. Although, it is likely that workplaces with strong Occupational Safety and Health (OSH) management were more inclined to participate in a work environment research project, and thereby increased the risk of selection bias at the workplace level. However, the high rate of participation (79%) among the participating workplaces included in the study is a strength adding to the generalizability of our findings.

The next step forward is to validate the AIWEI further at the organizational level. For example, how well an organization promotes and maintains age inclusiveness as collectively experienced by their workers. In particular, capturing systemic discrimination and systemic opportunities for development, as these factors demonstrated high consistency within organizations.

In conclusion, we provide a generic nine-item instrument that can be used as a quick and easy “environment check” for age-diverse organizations in how they are performing in relation to providing an age-inclusive work environment for both younger and older workers. Overall, we found support that our instrument has sound psychometric properties, using confirmatory factor analysis, measurement invariance analysis, and multilevel regressions as demonstrations of validity.

## Supplementary Material

igac066_suppl_Supplementary_Material
