# Supplementary material for: A Quick “Environment Check” for All Ages: Validating the New Age-Inclusive Work Environments Instrument
Source: Innov Aging. 2022 Oct 27;6(7):igac066. doi: 10.1093/geroni/igac066 (PMC9795836; doi:10.1093/geroni/igac066)
Supplement: igac066_suppl_Supplementary_Material [file igac066_suppl_supplementary_material.docx]

**Supplementary Table 1.** *The breakdown of personal and work demographics for the three age groups; younger (under 35), middle age (35 to 54), and older worker (55 and above).*

| Variables | Characteristics | Under 35  *n* (%) | 35 to 54  *n* (%) | 55 and above  *n* (%) |
| --- | --- | --- | --- | --- |
| Gender | Female | 535 (67.4%) | 896 (63.4%) | 376 (60.0%) |
|  | Male | 259 (32.6%) | 518 (36.6%) | 251 (40.0%) |
| Weekly work hours | 0-10 hours | 12 (1.5%) | 16 (1.1%) | 13 (2.1%) |
|  | 11-20 hours | 16 (2.0%) | 21 (1.5%) | 33 (5.3%) |
|  | 21-30 hours | 33 (4.1%) | 69 (4.9%) | 55 (8.8%) |
|  | 31-40 hours | 426 (53.3%) | 901 (63.5%) | 378 (60.8%) |
|  | More than 40 hours | 309 (38.7%) | 411 (28.9%) | 143 (23.0%) |
|  | Unspecified | 3 (0.4%) | 2 (0.2%) | 0 (0%) |
| Industry | Manufacturing | 62 (7.7%) | 97 (6.8%) | 20 (3.2%) |
|  | Supply of electricity, gas, heating and cooling | 35 (4.3%) | 38 (2.7%) | 12 (1.9%) |
|  | Water supply (sewage treatment, waste management and remediation) | 14 (1.7%) | 20 (1.4%) | 6 (1.0%) |
|  | Trade (repair of motor vehicles and motorcycles) | 35 (4.3%) | 30 (2.1%) | 6 (1.0%) |
|  | Real estate activities | 10 (1.2%) | 10 (0.7%) | 0 (0%) |
|  | Public administration | 399 (49.5%) | 420 (29.4%) | 132 (21.0%) |
|  | Training | 57 (7.1%) | 230 (16.1%) | 88 (14.0%) |
|  | Health and social care/social services | 61 (7.6%) | 289 (20.2%) | 170 (27.0%) |
|  | Culture, contentment, and leisure | 48 (6.0%) | 59 (4.1%) | 26 (4.1%) |
|  | Other service activities | 26 (3.2%) | 59 (4.1%) | 42 (6.7%) |

**Supplementary Table 2.** *Swedish to English translations for the Age Friendly Work Environment Instrument.*

| Item | Original Question (Swedish) | English Translation |
| --- | --- | --- |
| Age1 | Dessa frågor handlar om ifall det finns plats för alla på din arbetsplats – Är äldre personers erfarenheter uppskattade på din arbetsplats? | These questions are about whether there is room for everyone in your workplace – Are the experiences of older people appreciated in your workplace? |
| Age2 | Dessa frågor handlar om ifall det finns plats för alla på din arbetsplats – Är det vanligt att äldre medarbetare fungerar som mentorer för yngre på din arbetsplats? | These questions are about whether there is room for everyone in your workplace – Is it common for older employees to act as mentors for younger people in your workplace? |
| Age3 | Dessa frågor handlar om ifall det finns plats för alla på din arbetsplats – Har du noterat att yngre medarbetare diskrimineras på din arbetsplats? | These questions are about whether there is room for everyone in your workplace – Have you noticed that younger employees are discriminated against in your workplace? |
| Age4 | Dessa frågor handlar om ifall det finns plats för alla på din arbetsplats – Har du noterat att äldre medarbetare diskrimineras på din arbetsplats? | These questions are about whether there is room for everyone in your workplace – Have you noticed that older employees are discriminated against in your workplace? |
| SI1 | Dessa frågor handlar om ifall det finns plats för alla på din arbetsplats – Behandlas män och kvinnor som jämställda på din arbetsplats? | These questions are about whether there is room for everyone in your workplace – Are men and women treated as equals in your workplace? |
| SI2 | Dessa frågor handlar om ifall det finns plats för alla på din arbetsplats – Finns det utrymme för anställda av olika etnisk bakgrund och med olika religion? | These questions are about whether there is room for everyone in your workplace – Is there room for employees of different ethnic backgrounds and with different religions? |
| PD2 | Dessa frågor är om dig själv i relation till dina arbetsuppgifter – Har du möjlighet att lära dig något nytt genom ditt arbete? | These questions are about yourself in relation to your work tasks – Do you have the opportunity to learn something new through your work? |
| PD3 | De följande frågorna är om hur du uppfattar ditt arbete – Kan du använda ditt kunnande eller dina färdigheter i ditt arbete? | The following questions are about how you perceive your work – Can you use your knowledge or skills in your work? |
| PD4 | Dessa frågor är om dig själv i relation till dina arbetsuppgifter – Erbjuder ditt arbete möjligheter att utveckla dina färdigheter? | These questions are about yourself in relation to your work tasks – Does your job offer opportunities to develop your skills? |

**Supplementary Table 3.** *The pathways as part of the proposed model controlling for the impact of hours worked and industry upon workers’ level of burnout and engagement.*

| Pathways | Young Workers | | Middle Age and Older Workers | |
| --- | --- | --- | --- | --- |
|  | *β* | 95%CI  [LL, UL] | *β* | 95%CI  [LL, UL] |
| PSC → Inclusion | .57^***^ | [.31, .82] | .64^*^ | [.43, .85] |
| PSC → Discrimination | -.36^**^ | [-.59, -.14] | -.55^**^ | [-.83, -.23] |
| PSC → Development Opportunities | .43^**^ | [.15, .70] | .49^***^ | [.31, .67] |
| Inclusion → Engagement | .16^*^ | [.01, .31] | .19^***^ | [.12, .26] |
| Inclusion → Burnout | -.11^*^ | [-.21, -.01] | -.18^**^ | [-.25, -.11] |
| Discrimination → Engagement | -.04 | [-.14, .06] | -.05 | [-.10, .01] |
| Discrimination → Burnout | .08 | [-.00, .17] | .16^***^ | [.11, .22] |
| Development Opportunities → Engagement | .42^**^ | [.18, .66] | .45^***^ | [.38, .50] |
| Development Opportunities → Burnout | -.22^***^ | [-.30, -.14] | -.16^***^ | [-.21, -.11] |
| PSC → Inclusion → Engagement | -.23 | [-.90, .44] | .58 | [-.22, 1.38] |
| PSC → Inclusion → Burnout | -.72^**^ | [-1.20, -.25] | -.88^**^ | [-1.45, -.31] |
| PSC → Discrimination → Engagement | .02 | [-.43, .48] | -.53 | [-1.33, .27] |
| PSC → Discrimination → Burnout | .25 | [-.25, .75] | .55 | [-.16, 1.26] |
| PSC → Development Opportunities → Engagement | .45^*^ | [.11, .80] | .28 | [-.38, .95] |
| PSC → Development Opportunities → Burnout | .09 | [-.31, .48] | .04 | [-.21, .29] |

*Note*. Hours controlled for were 31-40 hours. Industries controlled for included Training, and Health and social care/social services.

^***^*p*<.001, ^**^*p*<.01, ^*^*p*<.05.
